# An analysis of 130 neuroendocrine tumors G3 regarding prevalence, origin, metastasis, and diagnostic features

**DOI:** 10.1007/s00428-021-03202-6

**Published:** 2021-09-09

**Authors:** Atsuko Kasajima, Björn Konukiewitz, Anna Melissa Schlitter, Wilko Weichert, Günter Klöppel

**Affiliations:** 1 Department of Pathology, Technical University Munich, Trogerstr. 18, 81675 Munich, Germany; 2 Member of the German Cancer Consortium (DKTK), Munich, Germany; 3 Department of Pathology, Universitätsklinikum Schleswig-Holstein, Christian-Albrechts-Universität Zu Kiel, Campus Kiel, Kiel, Germany

**Keywords:** Neuroendocrine tumors G3, Prevalence, Origin, Metastasis, Diagnosis

## Abstract

Limited data exist on high-grade neuroendocrine tumors (NETs G3) which represent a new category among neuroendocrine neoplasms (NEN). We analyzed NETs G3 in a consultation series regarding prevalence, origin, metastasis, and diagnostic problems. Based on the WHO classification of digestive system tumors, 130 NETs G3 (9%) were identified in 1513 NENs. NET G3 samples were more often obtained from metastatic sites (69%) than NET G1/G2 samples (24%). NET G3 metastases presented most frequently in the liver (74%) and originated from the pancreas (38/90, 42%), followed by the lung (9%), ileum (7%), stomach (3%), rectum (1%), and rare sites (2%) such as the prostate and breast. The primaries remained unknown in 15%. NETs G3 had a median Ki67 of 30% that distinguished them from NECs (60%), though with great overlap. The expression of site-specific markers, p53, Rb1, and SST2 was similar in NETs G3 and NETs G1/G2, except for p53 and Rb1 which were abnormally expressed in 8% and 7% of liver metastases from NET G3 but not from NET G1/G2. NETs G3 were frequently referred as NECs (39%) but could be well distinguished from NECs by normal p53 (92% versus 21%) and Rb1 expression (93% versus 41%) expression. In conclusion, NETs G3 are frequently discovered as liver metastases from pancreatic or pulmonary primaries and are often misinterpreted as NEC. p53 and Rb1 are powerful markers in the distinction of NET G3 from NEC. Rarely, carcinomas from non-digestive, non-pulmonary organs with neuroendocrine features may present as NET G3.

**Supplementary Information:**

The online version contains supplementary material available at 10.1007/s00428-021-03202-6.

## Introduction

The 2017 WHO classification of pancreatic neuroendocrine neoplasms (PanNENs) [1] and subsequently the 2019 WHO classification of digestive system tumors [2] stratified high-grade neuroendocrine neoplasms (NENs) into neuroendocrine tumor (NET) G3 and neuroendocrine carcinoma (NEC), mainly based on histology and genetic profile [3, 4]. While NETs G3 generally have retained the organoid pattern and the wild-type *TP53* and *Rb1* gene profile of the well-differentiated NENs, NECs display usually a disorganized pattern, significant cellular atypia, and genetic abnormalities in the *TP53* and *Rb1* genes. Since this stratification of high-grade digestive system NENs has clinical implications and seems also to be pertain to pulmonary NENs [5, 6] and other non-digestive organs [7–10], investigations on high-grade NENs, in particular the NETs G3, have received great attention. Despite these studies, data on NET G3, especially in non-pancreatic organs, are still scarce [11, 12]. Moreover, despite the WHO definition of criteria distinguishing NET G3 from NEC, diagnostic difficulties can occur in each case, notably in liver metastases.

We studied NETs G3 in a consultation series of 1513 NENs. The NETs G3 were identified using the criteria of the WHO classification 2019. As many NETs G3 presented as metastasis with a clinically unclear origin, the tumors were tested by a panel of markers with known site specificity, e.g., ISLET-1, CDX2, serotonin, and TTF-1. The special aims of this study were to investigate (1) the prevalence of NET G3 regarding origin and metastasis; (2) the characterization of NET G3 regarding Ki67 index and p53, Rb1, and SST2 expression; (3) the value of immunohistochemistry in the differential diagnosis of NET G3 versus NEC; and (4) the agreement between referral diagnosis and final diagnosis.

## Materials and methods

### Tissue assembling

We analyzed clinicopathological and immunohistochemical features in 1513 cases of a total of 1745 NENs, collected in our consultation center between April 2009 and April 2021, including NETs, NECs, and mixed neuroendocrine-non-neuroendocrine neoplasms (MiNENs). Two hundred thirty-two of 1745 NENs, encompassing cases of neuroendocrine microadenoma, insulinomatosis, glucagon cell hyperplasia and neoplasia, medullary thyroid carcinoma, pituitary adenoma, parathyroid neoplasm, Merkel cell carcinoma, and neuroendocrine precursor lesions (e.g. gastric neuroendocrine cell hyperplasia, pulmonary diffuse idiopathic neuroendocrine cell hyperplasia, duodenal G-cell hyperplasia, and neoplasia) as well as ectodermal NENs (paraganglioma, pheochromocytoma, olfactory neuroblastoma, and neuroblastic neoplasm), were excluded, because they were of no relevance for this study. The same consultation series was used to analyze mesenchymal neoplasms with neuroendocrine features as mimickers of NENs [13].

The patients’ age and sex and the origin of the samples are shown in Supplemental Table 1. Biopsies accounted for 44% (*N* = 645) of all specimens and were mostly obtained from the liver (*N* = 239).

The site of primary was determined in 1361 patients based on the organ tissue surrounding the tumor, the immunohistochemical findings indicating the origin of the lesion in case of metastases, and the information provided in the referral letters.

### Histopathological and immunohistochemical evaluation

Hematoxylin and eosin (HE) staining and periodic acid-Schiff (PAS) staining were done on 2-µm-thick sections from formalin-fixed paraffin-embedded tissues. Immunohistochemical stainings were performed using a fully automated slide preparation system (Benchmark XT, Ventanta/Roche, Arizona, USA). Details regarding the immunohistochemical stainings are given in Supplemental Table 2. Immunohistochemical expression of CDX2, ISLET-1, TTF-1, und serotonin was recorded as positive when more than 10% of the total tumor cells were stained. SST2 was evaluated using a previously reported four-tiered scoring system [14]. Briefly, positive tumors were scored 2 (membranous incomplete staining in less than 50%) or 3 (circumferential membranous staining in more than 50%) [14]. Abnormal p53 expression was defined as moderate to strong nuclear immunoreactivity in more than 20% of tumor cells [15]. Rb1 nuclear expression in less than 10% of tumor cells was defined as loss/abnormal expression [5]. Nuclear Ki67 labeling was counted in more than 500 tumor cells in the area with highest density (hot spot), and its percentage was given as Ki67 index (%). Immunohistochemistry of p53, Rb1, and SST2 was performed in 55% (72/130), 35% (46/130), and 66% (86/130) of all NET G3 cases, respectively. All cases were reviewed at least by two endocrine and pancreas pathology experts including AK, BK, MS, WW, and GK. When there was a disagreement on the diagnosis, consensus was reached after joint discussion at a multiheaded microscope. The final diagnosis of all cases (including the cases before 2017) was based on the current WHO classifications of tumors of the digestive system and tumors of the endocrine organs [1, 2]. All well-differentiated NENs, no matter what organ they came from, were classified and graded as NETs [16]. MiNENs were graded in low, intermediate, and high according to a recent proposal by La Rosa et al. [17] (Supplemental Table 1).

### Diagnostic features of NET G3 and NEC

**Fig. 1:**
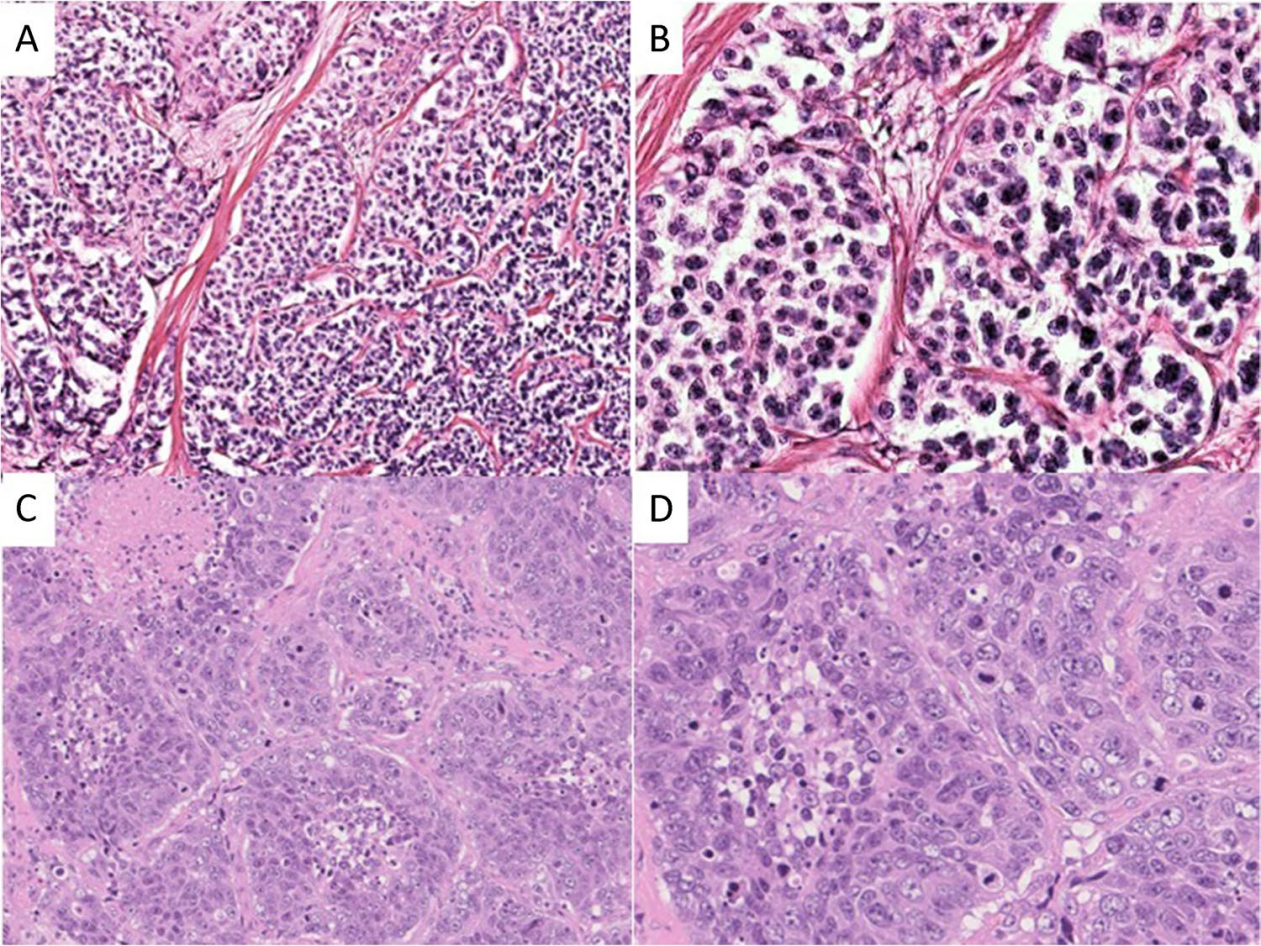
Histological images of NENs: NET G3 (**A**) showing an organoid, partly trabecular architecture, and (**B**) monomorphic cells. Large cell-type NEC (**C**) showing irregular nesting pattern with necroses and (**D**) pleomorphic cells with many mitoses and distinct nucleoli

NET was diagnosed, when the tumor showed an organoid architecture with either solid, solid-trabecular, or solid-glandular patterns (Fig. 1A), occasional intratumoral necroses, rather uniform nuclei with coarse granular chromatin and small nucleoli (Fig. 1B). The diagnosis of small cell-type NEC was based on a diffuse cell sheet pattern with geographic necroses. The cells had fusiform, occasionally also enlarged nuclei with finely granular chromatin, scant cytoplasm, and nuclear moulding. In the large cell-type NEC, the architecture was usually characterized by irregular nests (Fig. 1C) that often had necroses and occasionally showed peripheral palisading. The nuclei had prominent nucleoli with vesicular chromatin (Fig. 1D) [2].

### Consultation requests and referred diagnoses

From the referral letters, the requests of the consultation and the suggested or suspected diagnoses were extracted and categorized as follows: assessment of the neoplasms’ (1) classification into NET or NEC, (2) entity (i.e., NEN versus non-NEN); (3) primary site; (4) SST2 expression; and (5) other criteria such as infiltration into adjacent tissue, presence of lymphovascular invasion, margin status, and/or treatment options (Supplemental Table 1).

### Statistical analyses

JMP Pro version 14.0.0 software (SAS Institute, Inc., Cary, NC, USA) was used for all statistical analyses. A correlation coefficient was calculated by Spearman’s method. The sample number among multiple groups was compared using Pearson’s Chi-squared test or Fischer’s exact test. The Wilcoxon test was applied for the comparisons of continuous values or scores between multiple groups found to be non-normally distributed by Shapiro–Wilk test. A *p* value of < 0.05 was considered statistically significant.

## Results

### Prevalence of NET G3

One hundred thirty NETs G3 were identified among 1513 NENs, including 1103 NETs (71%), 255 NECs (17%), and 155 MiNENs (10%). NETs G3 accounted for 12% of all NETs and 9% of all NENs (Supplemental Table 1).

### Source of tumor samples

NET G3 samples were mainly of metastatic origin (90/130, 69%), with the liver (67/90, 74%) as leading site of metastasis, followed by lymph nodes (10/90, 11%) and various other organs (13/90, 14%). Most samples of primary NETs G3 (*N* = 40) were obtained from the pancreas (40%), followed by the lung (23%), the stomach (19%), and the rectum (8%) (Table 1). Single NETs G3 came from the duodenum/papilla of Vater (5%), ileum (3%), and one from the prostate (Table 1). In contrast to NETs G3, NETs G1/G2 (739/973, 76%) and MiNENs (120/155, 77%) were more often obtained from primary sites, while NECs were equally frequent in primary (125/255, 49%) and metastatic sites (130/255, 51%).

**Table.1:** Origin and prevalence of primary and metastatic neuroendocrine tumors G3

	Total NEN *N* (%)	NET G3 *N*	% Total number of NEN	% Total number of NET G3
Total N [%]	1513 (100)	130	9	100
Primary	1024 (68)	40	4	31
Metastasis	489 (32)	90	18	69
Pancreas	418 (28)	54	13	42
Primary	279 (67)	16	6	30
Metastasis	135 (33)	38	28	70
Stomach	174 (12)	10	6	8
Primary	165 (95)	7	4	70
Metastasis	9 (5)	3	33	30
Duodnum/PoV	108 (7)	2	2	2
Primary	97 (90)	2	3	100
Metastasis	11 (10)	0	0	0
Ileum	158 (10)	7	4	5
Primary	102 (65)	1	1	14
Metastasis	56 (35)	6	11	86
Appendix	79 (5)	0	0	0
Primary	77 (100)	0	0	0
Metastasis	0 (0)	0	0	0
Colon	82 (5)	1	1	1
Primary	75 (91)	1	1	100
Metastasis	7 (9)	0	0	0
Rectum	71 (5)	4	6	3
Primary	64 (90)	3	5	75
Metastasis	7 (10)	1	14	25
Lung	170 (11)	26	15	20
Primary	87 (51)	9	10	35
Metastasis	81 (49)	17	21	65
Other sites	101 (6)^a^	3^c^	3	2
Primary	76 (75)	1	1	33
Metastasis	25 (26)	2	8	67
Unknown	152 (10)^b^	23	15	18

Abbreviations: *NET* neuroendocrine tumor, *PoV* papilla of Vater. ^aI^including 5 esophagus, 12 biliary system, 2 liver, 4 kidney, 17 urinary tract, 15 prostate, 10 uterus, 8 ovary, 1 vagina, 9 breast, 3 presacral, 13 head and neck; ^b^All cases examined in metastatic site, ^c^1 prostate, 1 breast, 1 presacral

### Origin of metastasis

Based on the evaluation of the information on the tumor origin provided in the referral letters, and the expression patterns of site-specific markers conclusions on the origin of metastasis were possible in 337/489 (69%) cases, including 67 of 90 (74%) metastatic NETs G3. In the remaining 152 cases (including 23 NETs G3), the primary site of the tumor remained unknown. Table 2 summarizes the origin and prevalence of NET G3 liver metastases (*N* = 67) compared to that of NET G1/G2 (*N* = 135), NEC (*N* = 54), and MiNEN (*N* = 23). Most hepatic metastases of NET G3 could be assigned to the pancreas (49%, 33/67) and the lung (13%, 9/67). Only few metastases could be ascribed to the stomach, rectum, and other sites such as the breast and presacral region. The metastatic rate of pancreatic NETs G3 (49%) was higher than that of NETs G1/G2 (52/135, 39%), NECs (5/54, 9%), and MiNENs (7/23, 30%). Liver metastases from ileal NETs (5/67, 7%) were more commonly of NET G1/G2 grade (27/135, 20%) than of NET G3 grade. Among the hepatic metastases from lung NENs (*N* = 38), NEC metastases (17/38, 31%) exceeded NET G3 (9/38, 23%) and all the other NEN metastases. No NET G3 metastases could be ascribed to duodenal, appendiceal, and colonic primaries. This was not surprising, since NET G3 primaries in these organs were rare or absent (see below). Liver NEN metastases, whose primary remained unknown, mostly concerned NECs (27/54, 50%) followed by NETs G1/G2 (42/135, 31%), NETs G3 (14/67, 21%), and MiNENs (17/23, 4%) (Table 2). In metastases, in which the origin was uncertain, immunohistochemistry was performed. A pancreatic origin was strongly suggested by the expression of ISLET-1 in 88% (23/26) of NET G3 liver metastases, an ileal origin by the expression of CDX2 and serotonin in 80% (4/5) and 100% (5/5), respectively, and a pulmonary origin by TTF1 in 76% (6/7).

**Table.2:** Origin and prevalence of 279 hepatic metastases of neuroendocrine neoplasms

	Total *N* (%)	NET G3 *N* (%)	NET G1/G2 *N* (%)	NEC *N* (%)	MiNEN *N* (%)
Total N [%]	279 (100)	67 (100)	135 (100)	54 (100)	23 (100)
Pancreas	97 (35)	33 (49)	52 (39)	5 (9)	7 (30)
Stomach	5 (2)	3 (4)	0 (0)	0 (0)	2 (9)
Duodenum/PoV	2 (1)	0 (0)	0 (0)	0 (0)	2 (9)
Ileum	32 (11)	5 (7)	27 (20)	0 (0)	0 (0)
Appendix	0 (0)	0 (0)	0 (0)	0 (0)	0 (0)
Colon	5 (2)	0 (0)	1 (1)	0 (0)	4 (17)
Rectum	5 (2)	1 (1)	2 (1)	1 (2)	1 (4)
Lung	38 (14)	9 (23)	10 (7)	17 (31)	2 (9)
Other sites^a^	8 (3)	2 (3)^b^	1 (1)	4 (7)	1 (4)
Unknown	87 (31)	14 (21)	42 (31)	27 (50)	4 (17)

Abbreviations: *NET* neuroendocrine tumor, *NEC* neuroendocrine carcinoma, *MiNEN* mixed neuroendocrine-non-neuroendocrine carcinoma, *PoV* papilla of Vater, including primary sites of ^a^2 urinary tract, 2 prostate, 2 breast, 1 presacral, and 1 vagina, ^b^1 breast and 1 presacral

When the numbers of NET G3 primaries and NET G3 metastasis were considered together, it is obvious that most NETs G3 occurred in or derived from the pancreas (54/130, 42%), followed by the lung (26/130, 20%), stomach (10/130, 7%), ileum (7/130, 5%), rectum (4/130, 3%), and other sites including duodenum/papilla of Vater (2/130, 2%), presacral lesion, breast, and prostate (1/130, 1%, each). Eighteen percent (23/130) NETs G3 remained unknown regarding their origin (Table 1).

### Ki67 index and expression of p53, Rb1, and SST2

**Fig. 2:**
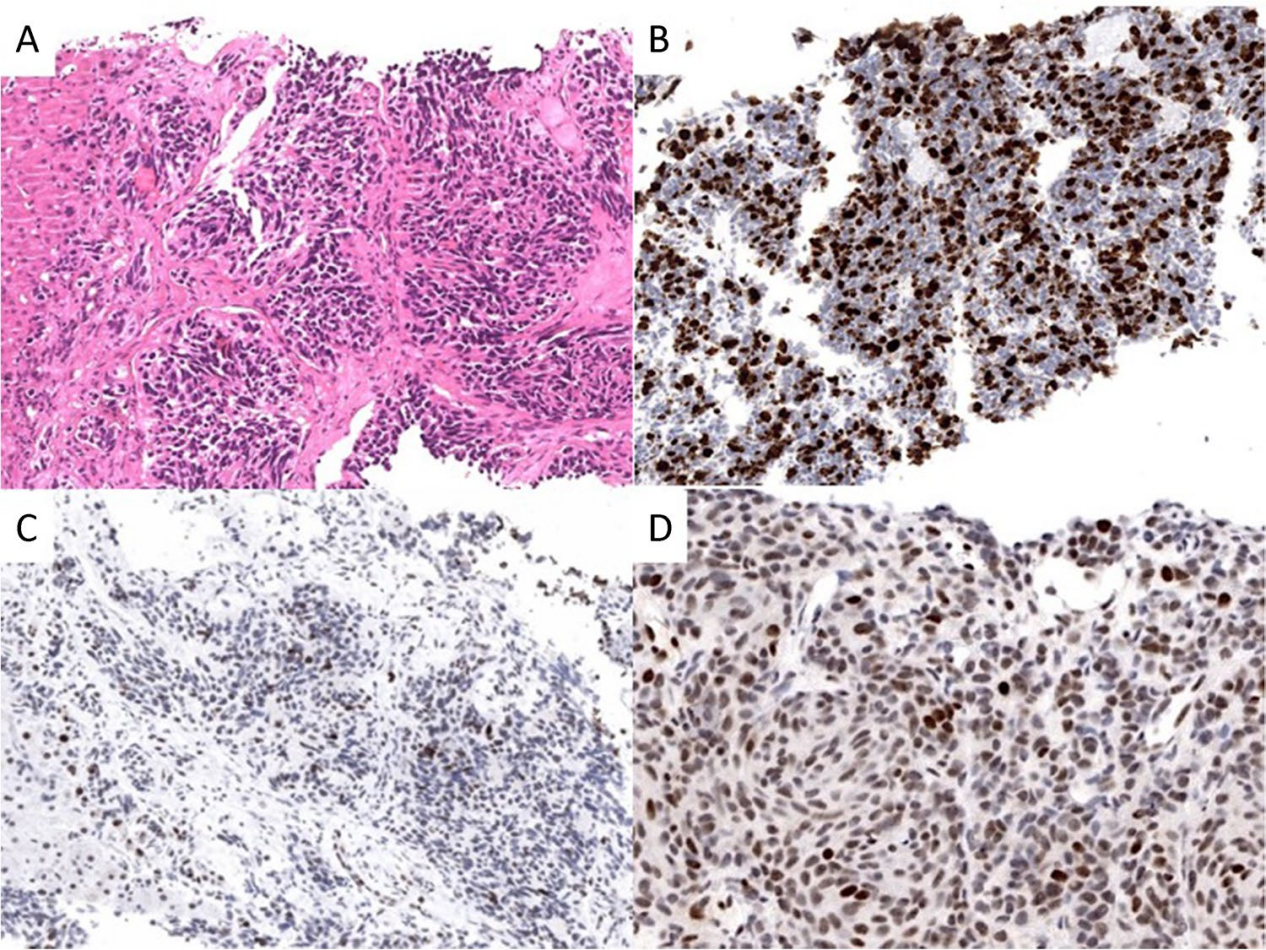
Histological and immunobiological images of liver biopsy specimen from a pulmonary neuroendocrine tumor G3. **A** Hematoxylin and eosin staining: solid tumor tissue infiltrating the liver. **B** Ki67 staining revealing a Ki67 index of 35%. **C** No abnormal expression of p53 showing only few tumor cells with weak staining. **D** Retained expression of nuclear retinoblastoma 1

**Fig. 3:**
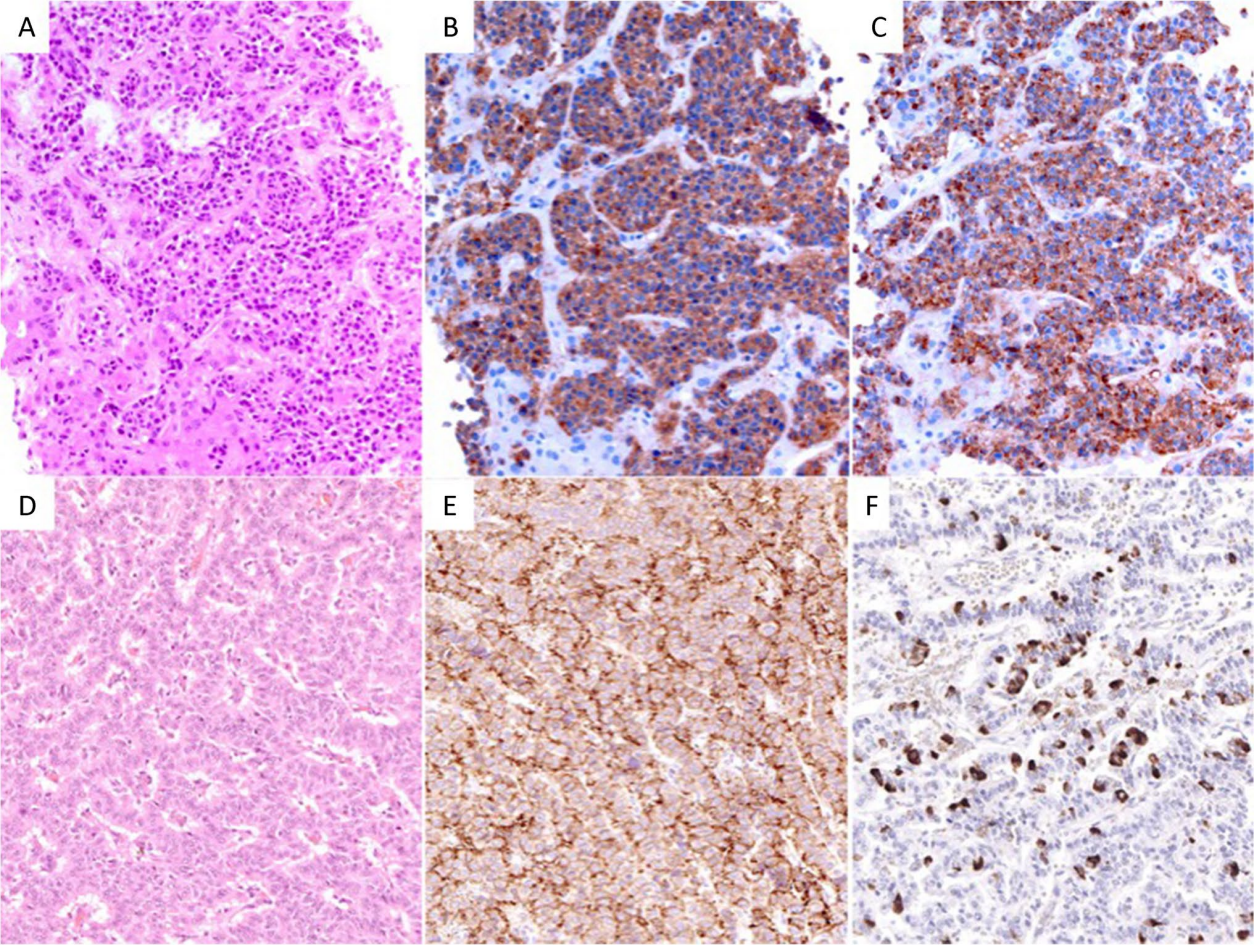
Histological and immunohistochemical images of NET G3 from rare sites. **A** Hematoxylin and eosin, **B** synaptophysin, and **C** chromogranin A staining of a breast NET G3. **D** Hematoxylin and eosin staining of a presacral NET G3 showing **E** strong membranous staining for somatostatin receptor 2 and **F** scattered cell positivity for serotonin

Ki67 index values of NET G3 ranged from 21 to 70% with a median of 30%. This median Ki67 index separated NETs G3 from NECs and MiNENs (*p* < 0.0001), while the range, as expected, showed a broad overlap between the three groups (Table 3). There was no difference between the median Ki67 index of NET G3 primaries versus metastases. The analysis of p53 and Rb1 revealed that these markers were not abnormally expressed in NET G1/G2 and only rarely in NET G3 (p53 8%, Rb1 7%) in contrast to NECs and MiNENs, in which abnormal p53 and Rb1 expression was high, reaching 80% and 60%, respectively (*p* < 0.0001 for both Table 3 and Fig. 2). In subsets of NETs G3 from non-pancreatic and non-pulmonary organs, abnormal p53 and Rb1 expression was observed in 10% and 20%, respectively. When we looked at NET G3 primaries and metastases separately, abnormal expression of p53 and Rb1 was only observed in specimens from metastases, mainly from the liver. SST2 expression rate was high in NETs G3 from the pancreas (70%) and from other digestive organs (59%, including the ileum, colon, and rectum) and low in NETs G3 from the lung (12%, Table 3). In the single NENs from the breast (Fig. 3a–c), prostate, and presacral regions (Fig. 3d–f), that corresponded to NETs G3, a normal expression of p53 and Rb1 and a strong SST2 expression (Fig. 3e) were observed. The breast NET G3, in addition, expressed ER and PgR, the presacral NET G3 serotonin (Fig. 3f), while the prostatic NET G3 failed to express NKX3.1.

**Table.3:** Immunohistochemical marker expression in NET G3 compared to other NEN types

Marker	Primary organ	Number examined		NET G3 *N* (%)	NET G1/G2 *N* (%)	NEC *N* (%)	MiNEN *N* (%)	*P* value NET G3 vs. NEC
Ki67 (%)	All organs	1450	Median (range)	30 (21–70)	2 (0.5–20)	60 (21–98)	50 (0.5–90)	< 0.0001
SST2	Pancreas	201	Negative	12 (30)	24 (18)	11 (83)	9 (82)	0.0003
			Positive	28 (70)	113 (82)	2 (15)	2 (18)	
	Lung	88	Negative	15 (88)	24 (56)	19 (68)	-	NS
			Positive	2 (12)	19 (44)	9 (32)		
	Other organs	408	Negative	12 (41)	41 (17)	70 (84)	48 (81)	< 0.0001
			Positive	17 (59)	196 (83)	13 (16)	11 (19)	
p53	All organs	372	Normal	66 (92)	87 (100)	28 (21)	27 (33)	< 0.0001
			Abnormal	6 (8)^a^	0	104 (79)	54 (67)	
Rb1	All organs	179	Normal	43 (93)	34 (100)	24 (41)	27 (66)	< 0.0001
			Loss	3 (7)^a^	0	34 (59)	14 (34)	

Abbreviations: *NET* neuroendocrine tumor, *NEC* neuroendocrine carcinoma, *MiNEN* mixed neuroendocrine-non-neuroendocrine neoplasm, *SST2* somatostatin receptor 2; ^a^only examined in metastasis

### Consultation requests

In total, 2120 requests were evaluated. The most frequently addressed requests concerned the differential diagnosis of NET versus NEC (34%), followed by the distinction of NEN from non-NEN (30%), the presumable origin of the primary (28%), the expression of SST2 (7%), and other minor issues such as the hormone expression (2%) (Supplemental Table 1). In NET G3, the issues of tumor origin (63%), differential diagnosis NEN versus non-NEN (39%), and NET versus NEC (37%) played the main role.

### Agreement between referral diagnosis and final diagnosis

In 1121 of 1513 NENs, a diagnosis was suspected or suggested by the submitting pathologist. The concordance rates between referral diagnosis and final diagnosis per tumor entity are listed in Supplemental Table 3. Lowest concordance was found in NETs G3 (20%) compared to all other entities, i.e., NET G1/G2 (66%), NECs (39%), and MiNENs (23%) (for details, see Supplemental Table 3). In NENs identified as NET G3, NECs (49%, 40/81) or NETs not otherwise specified (43%, 35/81) accounted for most inconsistent diagnoses (for details, see Supplemental Table 3). NET G3, on the other hand, was suggested as diagnosis in 10 NETs G2, 5 NECs, 7 MiNENs, and various other carcinomas including medullary thyroid carcinoma, adenocarcinoma, squamous cell carcinoma, acinar cell carcinoma, and SMARCB1-deficient neoplasm (data not shown). Of the 82 NETs G3 with an inconsistent diagnosis, 59 cases were from the years 2009 to 2017 (6.7 cases per year) and 23 cases from 2018 to 2021 (5.8 cases per year).

## Discussion

This study evaluated 1513 NENs and identified 130 NETs G3 of various origin, applying the criteria of histological differentiation and proliferative activity as defined in the 2019 WHO classification of digestive system tumors [16].

Previous studies on well-differentiated NENs with a Ki67 index above 20% and deriving from various organs identified the pancreas as the most frequent site of origin in 46–65% of the cases [9–11]. We confirm this data on NET G3 in the pancreas with a prevalence rate of 42%. Next in frequency in our cohort was the lung with 20%, a figure that is considerably higher than the 8% reported by Velayoudom-Cephise et al. [9]. The discrepancy is probably due to that our study included much more metastases than the other investigation. Other sites of origin that were included in previous studies [10, 11] as well as in our study were the stomach, the ileum, and the rectum. The available data (although difficult to extract from the literature [9, 10]) and our data indicate that gastric NETs G3 account for approximately 11%, which is higher than that of the ileum and rectum. In our cohort, NETs G3 from the ileum and the rectum accounted for about 5% and 3%, respectively. These low rates are in line with the rarity of respective cases reported [10, 18–20]. Among the very rare sites of NET G3 were the duodenum and papilla of Vater, ascending the colon, presacral region, breast, and prostate, with a rate of 1% each. Taken together, these prevalence data clearly indicate that the NETs G3 are unevenly distributed in the body. They parallel the prevalence of NETs G1/G2 in the pancreas, lung, stomach, and colon, but not in the ileum, appendix, and rectum. Noteworthy is this discrepancy in the ileum, as it again points to the special role that ileal NETs play among the remaining NETs in terms of histological, hormonal, and genetic features [21].

Among all NETs of the pancreas and the lung, the NETs G3 of this series were found in a percentage of 13% and 15%, respectively, and accounted for 9% of all NENs. These numbers are high compared with the data presented by Rindi (2.1%) and by Kim (9.7%) in pancreatic NENs [22, 23] and our own study on pulmonary NENs (12%) [5]. The reason for these discrepancies may be that, first, it must be assumed that NETs G3 are overrepresented in this study because of the case selection bias inherent in all consultation series. Second, included in the number of our NETs G3 are not only primaries but also the respective metastases which significantly exceeded (in a ratio of 1: 2–3) the number of primaries in the pancreas and the lung. The finding that metastases of pancreatic and pulmonary NETs G3 were more frequent than primaries suggests that some primary NETs G1/G2 progress to NETs G3 during metastatic spread. Similar conclusions may be drawn from other studies, which showed that there is often an evolution of the proliferative activity in NETs of the pancreas and the lung during the metastatic process [24, 25]. An exception from these observations in pancreas and lung NETs G3 seems to be the stomach NET G3, since the percentage of primary NETs G3 (mostly type 3 gastric NET) was higher (70%) than that of metastatic NETs G3. This is probably due to their early detection and removal by endoscopy [8].

The reason for the assumed progression of NET G1/G2 to NET G3 from the pancreas and the lung may be a genetic change that occurs during the metastasizing process, particularly to the liver. We found that the immunohistochemical expression of p53 and Rb1 was normal in NETs G1/G2 and commonly present in NECs. In NETs G3, abnormal expressions of p53 and Rb1 were found in 8% and 7%, respectively. The small difference between NETs G3 and NETs G1/G2 could be an indication that few NETs G3 probably have or develop *TP53* and *RB1* gene abnormalities that relate them to NECs. This hypothesis was also recently discussed by Pelosi et al. based on genetic studies on subsets of NETs and NECs in the gastrointestinal tract and the lung [26, 27]. Since we only discovered p53 and Rb1 abnormalities in NET G3 metastases and not in primaries, we suspect that these genetic abnormalities mainly develop during the metastasizing process, particularly to the liver.

It has been shown that SST2 is expressed in most digestive organ NETs including NETs G3 [15] while only in about 50% in lung NETs [5, 6, 15, 21, 28]. Here, we found that NETs G3 of digestive organ tumors express SST2 somewhat less frequent than the respective NETs G1/G2, but still distinctly more than NECs. In the lung, however, NETs G3 turned out to be the NENs with the lowest SST2 expression rate (13%), since not only NET G1/G2 (44%) but also NECs (32%) express SST2 at a distinctly higher rate [29].

Infrequent sites of NET G3 recorded in our series and/or reported in the literature included Vater papilla, ascending colon, and presacral region. No NETs G3 have so far been observed by us and others in the esophagus, jejunum, appendix, and hepatobiliary tract [10, 18]. We also diagnosed a NET G3 in a liver metastasis from primary tumors of the prostate and breast, respectively. In both cases, the metastases presented as solid tumor tissue with endocrine appearance. In the case of the breast metastasis, its origin was revealed by the positivity for the markers ER and PgR. In the case of the other metastasis, an origin from the prostate was confirmed by the subsequent resection of a prostate carcinoma which had a solid neuroendocrine component negative for the prostate marker NKX3.1. Since in the prostate and the breast the classification of NENs does not include a NET G3 category [30, 31], the discussion of the presented neuroendocrine breast and prostate liver metastases with NET G3 morphology serves only to point out that metastases with NET G3 features can occur in the liver, that derive from primaries in organs, in which NETs G3 have so far not been observed and discussed.

In the consultation requests, the issue of tumor origin played an important role in the tumors identified as NET G3, since most NET G3 samples came from metastatic sites. Helpful in solving this issue was the use of a panel of transcription factors known as markers for gastroenteropancreatic or pulmonary NETs such as CDX2 [32], ISLET-1 [33, 34], TTF-1 [35], and serotonin [36–38]. This differential testing also revealed that NETs G3 are similar, or identical, in their expression rates to NET G1/G2. Noteworthy is that serotonin was a very indicative marker for ileal NETs, when it was diffusely positive and accompanied by a nested histology with peripheral palisading of the tumor cells.

Another frequent request was the differentiation of NET G3 from NEC, MiNEN, and occasionally NET and other non-neuroendocrine carcinomas. Well- or poorly differentiated histology, i.e., organoid versus diffuse architecture, rare and small necrosis versus common geographic necrosis and moderate versus high-grade cellular atypia are the main defining criteria for NET versus NEC and most MiNEN in our study, as well as in other recent studies [10, 11, 23]. These criteria found a high degree of agreement (84%) by pathology experts, who re-evaluated 196 high-grade NENs with regard to their distinction in NET G3 and NEC [11]. However, assessment of histological differentiation may be difficult in biopsies [39] and occasionally also in resection specimens [40]. This is also true for the Ki67 index, which as a median value is a guiding parameter and significantly separates NET G3 from NEC (30% versus 60% in our study). The individual values, however, may overlap with the values of the other category. In these difficult cases, as could be demonstrated in our series, p53 and Rb1 immunohistochemistry was helpful for distinguishing NETs G3 from NECs. These data that are in line with previous studies in the pancreas [15, 40] and the lung [5, 6] also include NETs G3 from other organs (see Table 3) which, except for a case report on an ileal NET G3 [19], have so far not been examined.

Matching the referral diagnoses with the final diagnoses revealed that the distinction of NET G3 from NEC achieved the lowest concordance. This concerned particularly the consultation cases, received till 2017, and may be explained by the definition of NEC provided in the 2010 WHO classification of digestive tumors, in which NECs were distinguished from NETs by a Ki67 index above 20%. Thus, all NENs with a Ki67 index > 20% were classified as NEC. This problem was only solved with the release of the 2017 WHO classification of endocrine tumors, in which the NET G3 category was defined for the pancreatic NENs. Since then, the NET-NEC issue is slowly losing its importance as a diagnostic problem, as we have noticed a slight decrease in the number of inconsistent NET-NEC diagnoses in the years 2018 to 2021 compared to the period of 2009 to 2017 (5.8 versus 6.7 cases per year).

In conclusion, the 130 NETs G3 identified in our consultation series mostly originated from the pancreas and lung and showed a high metastatic potential that often manifested itself as metastasis to the liver. It seems that a low number of NET G3 acquire an abnormal expression of p53 and RB1 during the metastatic process, indicating mutations in the respective genes. Our case review also showed that the potential of NETs to develop into NETs G3 is site-specific, with very few NETs G3 in the ileum where NETs G1/G2 are otherwise frequent. Primaries or metastases from NENs with the features of NET G3 can occasionally emerge from organs that are known to develop neoplasms with neuroendocrine differentiation, which however are difficult to classify and compare to the NENs of the digestive system or lung [30, 31]. The main diagnostic problem encountered in our consultation series, particularly in biopsies, is the distinction of NET G3 from NEC, for which the application of the markers p53 and Rb1 proved to be very helpful.

## Supplementary Information

Below is the link to the electronic supplementary material.

Supplementary file1 (XLSX 22 KB)
